# Investigation of New Tsallis-Based Equation to Predict Shear Stress Distribution in Circular and Trapezoidal Channels

**DOI:** 10.3390/e21111046

**Published:** 2019-10-26

**Authors:** Zohreh Sheikh Khozani, Wan Hanna Melini Wan Mohtar

**Affiliations:** Department of Civil Engineering, Faculty of Engineering & Built Environment, Universiti Kebangsaan Malaysia, UKM Bangi 43600, Selangor, Malaysia; hanna@ukm.edu.my

**Keywords:** Tsallis entropy, circular channel with flat bed, trapezoidal channel, Shannon entropy, shear stress distribution prediction

## Abstract

In this study, the entropy concept is employed to estimate the shear stress distribution in a circular channel with flat bed and trapezoidal channel. Using the principle of maximum entropy, the shear stress distribution is derived by maximizing the Tsallis entropy by assuming averaged shear stress as a random variable. The derived shear stress equation can describe the variation of shear stress along the wetted perimeter of channel. The developed model of shear stress distribution is tested with some credible experimental data and is also compared with equations obtained by other researchers based on the Shannon entropy concept. The present model has shown good agreement with the observed data and performed better than the Shannon-based model in both cross-sections with better results of several computed quantitative criteria. The model precision in estimating shear stress in the trapezoidal channel with mean root mean square error (RMSE) of 0.0158 was higher than the circular channel with flat bed with RMSE of 0.0679.

## 1. Introduction

The shear stress distribution of a channel influences important benthic processes such as sediment transport, deposition, and channel morphology. The boundary shear stress distribution along the wetted perimeter of a smooth channel depends on the channel aspect ratio and the structure of secondary flow cells. A considerable amount of experimental work has been carried out on the distribution of shear stress in the open channel e.g., [1,2] in the rectangular open channel, by [3,4,5] in the trapezoidal channel, and [6,7] in circular cross section. The presentation of boundary shear stress in terms of bed and sidewall subsections has been extensively described through analytical methods presented by [8,9,10,11,12]. Zarrati et al. [10] applied a simplified streamwise vorticity equation that includes secondary Reynolds stresses and proposed a semi-analytical model to predict shear stress distribution in simple and compound rectangular and trapezoidal channels. Although their proposed model performed well in predicting the shear stress distribution, the calculation of shear stress requires a number of equations, which is time-consuming [13]. The increasing use of new numerical methods based on a soft computing approach offers an alternative in the determination of shear stress distribution with more time effective. The utilization of such approach was successfully conducted to forecast in various hydraulics-related phenomena such as the estimation of sediment transport [14,15,16], mean wall or bed shear stress in rectangular channels with smooth and rough boundaries [12,17,18], and apparent shear stress in compound channels [19]. In predicting the shear stress distribution around wetted perimeter in circular channel, Sheikh Khozani et al. [20] applied gene expression programming (GEP) and extracted an equation to calculate the shear stress, which only related to Reynolds number and the non-dimension parameter *y*/*P* (where *P* is whole wetted perimeter and *y* is the transversal coordinate). Sheikh Khozani et al. [21] also used the randomized neural network (RNN) to predict shear stress in circular channels without sediment and extracted a matrix-based equation. They investigated the impact of different input variables in estimating shear stress distribution and finally introduced the best input variables for a high modelling performance.

The entropy concept is one of the analytical methods applied by Chiu [22] to forecast velocity and shear stress, where they employed the Shannon entropy. After the work of Chiu [22], many researchers utilized different entropy models for predicting different phenomena [13,23,24,25,26,27,28,29]. Mirauda et al. [30] applied the entropy concept to find a simple presentation of suspended sediment concentration. Sterling and Knight [25] applied the Shannon entropy to predict shear stress in circular and trapezoidal channels and noted that although their model performed well, it needs more investigation. Sheikh and Bonakdari [31] used the Shannon entropy concept to estimate shear stress in circular channels. They could present simpler and more accurate equations than the representation proposed by [25]. Sheikh Khozani and Bonakdari [32] applied the Renyi entropy approach to forecast shear stress distribution in circular channels and extracted equations to compute the wall and bed shear stress along wetted perimeter. The Tsallis entropy is a generalization of the Shannon entropy that was used by [33] to predict bed-load layer thickness in open channels, and a comparison between the results of Tsallis with the Shannon entropy-based expression was made. Bonakdari et al. [34] utilized the Tsallis entropy to estimate shear stress distribution in circular, rectangular, and compound channels. They presented equations based on maximizing the entropy function that calculated shear stress using Lagrange multipliers, which need to solve a set of explicit relations. To calculate the proposed equations of shear stress prediction by Bonakdari et al. [26], the Lagrange multipliers should be computed by using two explicit equations, whereby solving these equations is complicated.

Circular and trapezoidal cross-sections are usually installed in sewer channels and irrigation channels, respectively. Although the Tsallis entropy method is able to extract equations, the procedures are complicated to solve due to the complexity in calculating the Lagrange multipliers. Therefore, deriving simpler and more accurate equations applicable to the common channel cross sections is necessary. The objective of this paper to apply the Tsallis entropy and define new parameters to extract simpler equations for estimating the shear stress distribution in circular and trapezoidal channels. In achieving the objective, the shear stress distribution derived (based on the Tsallis entropy) is verified using credible experimental data produced by Knight and Sterling [6] (in circular cross section) and Yuen [5] (for the trapezoidal cross section). Finally, the performance of the proposed Tsallis-entropy based model is compared with the results from Shannon entropy-based equations, which were represented by [25].

## 2. Derivation of Shear Stress Using Tsallis Approach

Procedural derivation of shear stress using the Tsallis entropy follows seven main steps. The first one is to define the Tsallis entropy before specifying the constraints. Next is maximizing the entropy, then deriving the probability distribution of shear stress. Finding the maximum entropy is the fifth procedure, followed by the determination of Lagrange multipliers and the derivation of the shear stress distribution makes up the last element. The temporally averaged shear stress τ is denoted as a random variable with a probability density function (PDF), $f_{\tau }$. The mathematical description of each described step is presented herein.

*Step 1. Definition of Tsallis entropy*: The shear stress described τ, or of f(τ), H(τ) using the Tsallis entropy [35] is expressed as:

(1) $$H\left(\tau \right)=H\left[f\left(\tau \right)\right]=\frac{1}{m-1}\int_{0}^{\tau_{max}}f\left(\tau \right)\left(1-{\left[f\left(\tau \right)\right]}^{m-1}\right)d\tau$$

where *m* is the entropy index and $\tau_{max}$ represents the maximum shear stress. Equation (1) provides the uncertainty value about f(τ), calculated as $\left[\left(1-{\left[f\left(\tau \right)\right]}^{m-1}\right)\right]/\left(m-1\right)$ or the averaged information content of sample τ. As such, the derivation of f(τ) is critical to be first derived through maximizing H(τ), whereby the H(τ) is subjected to the specified constraints. The principle of maximum entropy (POME) as outlined in Jaynes [36,37] allows a determination of f(τ) with specific bias to known parameters (of shear stress) instead of the unknowns. The deployment of POME requires specific information on shear stress, expressed as the constraints, leading to the determination of the most suitable f(τ) with the maximum entropy or uncertainty.

*Step 2. Specification of Constraints*: In deriving the shear stress, the total probability law specific constraints, which are mandatory to satisfy the f(τ), are expressed as:

(2) $$C_{1}=\int_{0}^{\tau_{max}}f\left(\tau \right)d\tau =1$$

(3) $$C_{2}=\int_{0}^{\tau_{max}}\tau f\left(\tau \right)d\tau =\bar{\tau }$$

where $C_{1}$ defines the total probability law and $C_{2}$ presents the mean shear stress.

*Step 3. Maximization of entropy*: In achieving the least biased f(τ), the Tsallis entropy as described in Step 1 is maximized using the method of Lagrange multipliers as:

(4) $$\begin{array}{c} L=\int_{0}^{\tau_{max}}\left(\frac{1}{m-1}f\left(\tau \right)\left(1-{\left(f\left(\tau \right)\right)}^{m-1}\right)\right)d\tau +\lambda_{0}\left(\int_{0}^{\tau_{max}}f\left(\tau \right)d\tau -1\right)\\ +\lambda_{1}\left(\int_{0}^{\tau_{max}}\tau f\left(\tau \right)d\tau -\bar{\tau }\right) \end{array}$$

Note that the f(τ) is subjected to Equations (2) and (3). Differentiating Equation (4) based on the calculus of variation and by setting the derivation value as 0, the entropy-based f(τ) is presented as:

(5) $$f\left(\tau \right)={\left[\frac{m-1}{m}\left(\frac{1}{m-1}+\lambda_{0}+\lambda_{1}\tau \right)\right]}^{1/\left(m-1\right)}.$$

The f(τ) in Equation (5) has two unknown Lagrange multipliers, which can be calculated using Equations (2) and (3). Incorporating Equation (5) into Equation (2) leads to the expression of:

(6) $$\int_{0}^{\tau_{max}}{\left[\frac{m-1}{m}\left(\frac{1}{m-1}+\lambda_{0}+\lambda_{1}\tau \right)\right]}^{1/\left(m-1\right)}d\tau =1$$

Solving Equation (6) resulted in:

(7) $${\left(\frac{1}{m-1}+\lambda_{0}+\lambda_{1}\tau_{max}\right)}^{\frac{m}{m-1}}-\left(\frac{1}{m-1}+\lambda_{0}\right)=\lambda_{1}{\left(\frac{m}{m-1}\right)}^{\frac{m}{m-1}}$$

The next procedure is to substitute Equation (5) in Equation (3) to obtain:

(8) $$\int_{0}^{\tau_{\max}}\tau {\left[\frac{m-1}{m}\left(\frac{1}{m-1}+\lambda_{0}+\lambda_{1}\tau \right)\right]}^{1/\left(m-1\right)}\;d\tau =\bar{\tau }$$

The solution of Equation (8) results in:

(9) $$\begin{array}{c} \tau_{max}{\left(\frac{1}{m-1}+\lambda_{0}+\lambda_{1}\tau_{max}\right)}^{\frac{m}{m-1}}+\frac{m-1}{2m-1}\left(\frac{1}{\lambda_{1}}\right){\left(\frac{1}{m-1}+\lambda_{0}+\lambda_{1}\tau_{max}\right)}^{\frac{2m-1}{m-1}}\\ -\frac{m-1}{2m-1}\left(\frac{1}{\lambda_{1}}\right){\left(\frac{1}{m-1}+\lambda_{0}\right)}^{\frac{2m-1}{m-1}}=\lambda_{1}\bar{\tau }{\left(\frac{m}{m-1}\right)}^{\frac{m}{m-1}} \end{array}$$

For simplification, let $\left(1/m-1\right)+\lambda_{0}=\lambda_{*}$; therefore, Equations (8) and (9) can be rewritten, respectively as:

(10) $${\left(\lambda_{*}+\lambda_{1}\tau_{max}\right)}^{\frac{m}{m-1}}=\lambda_{1}{\left(\frac{m}{m-1}\right)}^{\frac{m}{m-1}}+{\left(\lambda_{*}\right)}^{\frac{m}{m-1}}$$

(11) $$\begin{array}{ll} \tau_{max}{\left(\lambda_{*}+\lambda_{1}\tau_{max}\right)}^{\frac{m}{m-1}} & +\frac{m-1}{2m-1}\left(\frac{1}{\lambda_{1}}\right){\left(\lambda_{*}+\lambda_{1}\tau_{max}\right)}^{\frac{2m-1}{m-1}}-\frac{m-1}{2m-1}\left(\frac{1}{\lambda_{1}}\right){\left(\lambda_{*}\right)}^{\frac{2m-1}{m-1}}\\ & =\lambda_{1}\bar{\tau }{\left(\frac{m}{m-1}\right)}^{\frac{m}{m-1}} \end{array}$$

Equations (10) and (11) are implicit in the Lagrange multipliers, so solving them becomes somewhat difficult.

The cumulative f(τ) is determined by the integration of Equation (5) from 0 to τ as:

(12) $$F\left(\tau \right)=\int_{0}^{\tau }f\left(\tau \right)d\tau =\int_{0}^{\tau }{\left[\frac{m-1}{m}\left(\lambda_{*}+\lambda_{1}\tau \right)\right]}^{\frac{1}{m-1}}d\tau =\frac{1}{\lambda_{1}}\left(\frac{m}{m-1}\right){\left[\frac{m-1}{m}\left(\lambda_{*}+\lambda_{1}\tau \right)\right]}^{\frac{m}{m-1}}.$$

Equation (12) can be rewritten as follows:

(13) $$\tau =-\frac{\lambda_{*}}{\lambda_{1}}+\frac{1}{\lambda_{1}}\left(\frac{m}{m-1}\right){\left[\lambda_{1}F\left(\tau \right)+{\left(\frac{m-1}{m}\lambda_{*}\right)}^{\frac{m}{m-1}}\right]}^{\frac{m-1}{m}}$$

The newly derived τ (Equation (13)) has a relationship of a probability quantile. By substituting Equation (5) in Equation (1), the maximum Tsallis entropy or uncertainty of shear stress can be determined based on the following expression:

(14) $$H=\frac{1}{m-1}\left(\tau_{max}+{\left(\frac{m-1}{m}\right)}^{m/m-1}\left(\frac{1}{\left(2m-1\right)\lambda_{1}}\right){\left(\lambda_{1}\tau_{max}\right)}^{2m-1/m-1}\right)$$

*Step 4. Derivation of shear stress distribution*: Let the wetted perimeter of channel be denoted as *P*. The shear stress values at the distance are measured from the interface of water y and air in left side of channel to any point of wetted perimeter. The cumulative F(τ) can then be presented as the ratio of shear stress (at the point of consideration) and the wetted perimeter [26], expressed as:

(15) $$F\left(\tau \right)=\frac{y}{P}$$

where F(τ) represents the cumulative distribution function (CDF). The F(τ) is linear in terms of τ if the relationship of τ and y is linear. Since Equation (15) serves as the fundamental hypothesis to derive the entropy-based shear stress distribution, assessment of the validity is necessary. The work of [35] successfully validated the hypothetical Equation (15).

The parameter f(τ) is calculated by differential procedure of Equation (15) with respect to τ as follows:

(16) $$f\left(\tau \right)=\frac{dF\left(\tau \right)}{d\tau }=\frac{1}{P}\frac{dy}{d\tau }orf\left(\tau \right)={\left(P\frac{d\tau }{dy}\right)}^{-1}$$

With substituting Equation (5) in Equation (16), the below relation obtained as:

(17) $${\left[\frac{m-1}{m}\left(\lambda_{*}+\lambda_{1}\tau \right)\right]}^{1/\left(m-1\right)}d\tau =\frac{dy}{P}$$

Integrating Equation (17) results in:

(18) $$\tau =-\frac{\lambda_{*}}{\lambda_{1}}+\frac{1}{\lambda_{1}}\left(\frac{m}{m-1}\right){\left[\lambda_{1}\frac{y}{P}+{\left(\frac{m-1}{m}\lambda_{*}\right)}^{\frac{m}{m-1}}\right]}^{\frac{m-1}{m}}$$

**Figure 1:**
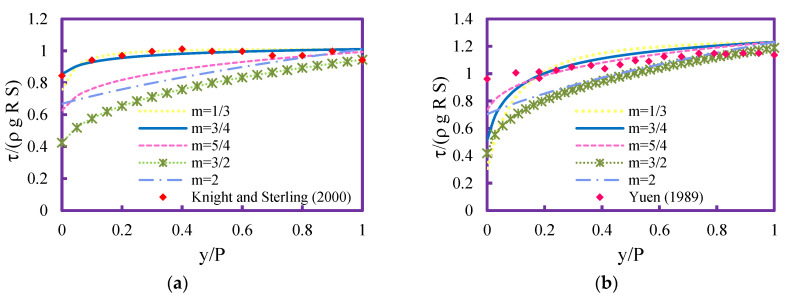
Dimensionless shear stress distribution for various *m* values for (**a**) circular channel, and (**b**) trapezoidal channel.

*Step 5. Derivation of shear stress distribution*: The determination of the entropy index m is obtained through calculation of shear stress distribution based on various m values from 1/3 to 3 as presented in Figure 1. The calculated τ shows that the parameter m significantly influenced the magnitude of shear stress distribution. Using m>3/4, the predicted τ is not close to the experimental data and by lowering the values for entropy index were able to estimate better agreement to experimental data. Although m = 1/3 presented good results, m = 3/4 fitted the observations best in both figures. Therefore, the entropy index value was selected as 3/4.

*Step 6. Reparameterization*: Reparameterization of the Lagrange multipliers allows the determination of shear stress with only one parameter. Therefore, if the shear stress reaches maximum value, then $f\left(\tau_{max}\right)=1$ and Equation (13) can be expressed as:

(19) $$\tau_{max}=-\frac{\lambda_{*}}{\lambda_{1}}+\frac{1}{\lambda_{1}}\left(\frac{m}{m-1}\right){\left[\lambda_{1}+{\left(\left(\frac{m-1}{m}\right)\lambda_{*}\right)}^{\left(\frac{m}{m-1}\right)}\right]}^{\left(\frac{m-1}{m}\right)}.$$

To obtain a simpler equation, a new dimensionless parameter is introduced as:

(20) $$G=\frac{\lambda_{1}\tau_{max}}{\lambda_{*}+\lambda_{1}\tau_{max}}$$

Dividing Equation (18) by Equation (19) and substituting dimensionless parameter *G*, one obtained as:

(21) $$\frac{\tau }{\tau_{max}}=1-\frac{1}{G}\left(1-{\left\{{\left(1-G\right)}^{\frac{m}{m-1}}+\left[1-{\left(1-G\right)}^{\frac{m}{m-1}}\right]\left(\frac{y}{L}\right)\right\}}^{m-1/m}\right)$$

It is noted when *y* = 0, then F(τ)=0; therefore, Equation (21) is reduced to:

(22) $$0=1-\frac{1}{G}\left(1-{\left(1-{\left(\left(\frac{m}{m-1}\right)\left(\frac{G}{1-G}\right)\tau_{max}\right)}^{m/m-1}\right)}^{m-1/m}\right)$$

Rearranging Equation (22), we obtained:

(23) $${\left(\frac{m}{m-1}\times \frac{G}{1-G}\tau_{max}\right)}^{m/m-1}=1-{\left(1-G\right)}^{m/m-1}$$

Equation (23) expresses $\tau_{max}$ as a function of *G* parameter for a given index entropy *m*. If m = 3/4 is taken, the maximum shear stress is:

(24) $$\tau_{max}=3\left(\frac{G-1}{G}\right){\left[1-{\left(1-G\right)}^{-1/3}\right]}^{-3}$$

**Figure 2:**
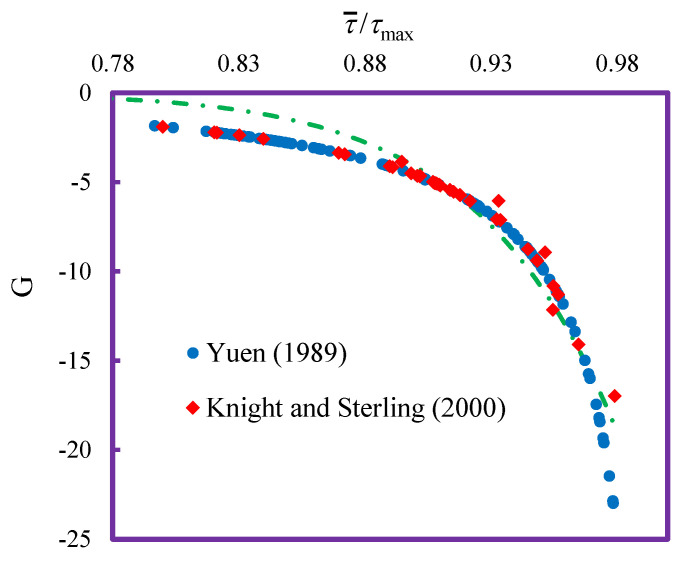
Relationship between entropy index, G and $\frac{\bar{\tau }}{\tau_{max}}$.

In Equation (21), the Lagrange multipliers are replaced with the dimensionless entropy parameter G permitting the value of shear stress with only one G. Parameter G can be used as the uniformity indicator of f(τ), corresponding to the maximum shear stress. Since the Lagrange multipliers are related to mean and maximum shear stress and G parameter is related to Lagrange multipliers, then we can obtain a relationship between G and $\frac{\bar{\tau }}{\tau_{max}}$. The value of G calculated based on Equation (20) is permissible by solving the Lagrange multipliers using Equations (10) and (11). The calculated G is then plotted against the relative mean shear stress and maximum shear stress ($\frac{\bar{\tau }}{\tau_{max}}$.), here shown in Figure 2.

Using regression, a simple equation was obtained to express relationship between G parameter and $\frac{\bar{\tau }}{\tau_{max}}$ as below:

(25) $$G=-27.3195{\left(\frac{\bar{\tau }}{\tau_{max}}\right)}^{17.9177}$$

Now, the shear stress distribution can be calculated using Equation (18), which only needs to calculate the dimensionless *G* parameter, and we are free of computing Lagrange multipliers.

By considering m=3/4, the Lagrange multipliers were obtained by solving the nonlinear Equations (10) and (11) for a specific τ and $\tau_{max}$. The calculated the Lagrange multipliers and the value of G parameter computed by Equations (20) and (25) are given in Table 1. It can be seen that the difference between values of two equations are less than 0.3, and it can be acceptable that we use Equation (25) instead of Equation (20).

For computing $\tau_{max}$ and $\tau_{mean}$, to calculate *G* parameter, the relations presented by Knight et al. [38] were used, which are expressed as follows:

(26) $$\frac{\tau_{mean\left(w\right)}}{\rho gRS}=0.01\%SF_{w}\left(1+P_{b}/P_{w}\right)$$

(27) $$\frac{\tau_{mean\left(b\right)}}{\rho gRS}=\left(1-0.01\%SF_{w}\right)\left(1+P_{b}/P_{w}\right)$$

(28) $$\frac{\tau_{max\left(w\right)}}{\rho gRS}=0.01\%SF_{w}\left[2.0372{\left(P_{b}/P_{w}\right)}^{0.7108}\right]$$

(29) $$\frac{\tau_{max\left(b\right)}}{\rho gRS}=\left(1-0.01\%SF_{w}\right)\left[2.1697{\left(P_{b}/P_{w}\right)}^{-0.3287}\right]$$

where $\tau_{mean\left(w\right)}$ and $\tau_{mean\left(b\right)}$ are the averaged shear stress at the wall and bed, respectively, ρ is the fluid density, *g* is the gravitational acceleration, *R* denotes the hydraulic radius, *S* is the bed slope, and *P_b_* and *P_w_* are the wetted perimeter of the bed and wall of the channel, respectively. The maximum shear stress is determined both at the wall $\tau_{max(w)}$ and bed $\tau_{max(b)}$ and *%SF_w_* is the percentage of shear force carried by walls, which can be calculated based on the following:

(30) $$\%SF_{w}=C_{sf}exp\left(-3.23\;log\left(P_{b}/C_{2}P_{w}+1\right)+4.6052\right)$$

where $C_{sf}=1.0$ for $\frac{P_{b}}{P_{w}}\leq 4.374$, unless $C_{sf}=0.5857{\left(P_{b}/P_{w}\right)}^{0.28471}$ and in subcritical flow $C_{2}=1.5$; It is noted that Equations (26) to (30) were used in the studies of [13,25,26].

## 3. Shannon Entropy

The prediction of localized shear stress at the wall $\tau_{w}$ and bed $\tau_{b}$ in the circular channel with flat bed and trapezoidal channels was obtained using the Shannon entropy as described by Sterling and Knight [25]. Both parameters of $\tau_{w}$ and $\tau_{b}$ are described as:

(31) $$\tau_{w}=\frac{1}{\lambda_{w}}\left[1+\left(e^{\lambda_{w}\tau_{max(w)}}-1\right)\frac{2\left(y-y_{w}\right)}{P_{w}}\right]y_{w}<y<\frac{P_{w}}{2}$$

(32) $$\tau_{b}=\frac{1}{\lambda_{w}}\left[1+\left(e^{\lambda_{b}\tau_{max(b)}}-1\right)\frac{2\left(y-y_{w}\right)}{P_{b}}\right]\frac{P_{w}}{2}<y<\frac{P_{w}}{2}+y_{w}$$

where *y_w_* = 0.005 m, $P_{b}$ is the wetted perimeter corresponding to the bed of the channel, $P_{w}$ is the wetted perimeter corresponding to the wall of the channel, and the Lagrange multipliers, λ, can be estimated as:

(33) $$\lambda =\left[\frac{\tau_{max}e^{\lambda \tau_{max}}}{e^{\lambda \tau_{max}}-1}-\rho gRS\right]$$

## 4. Data Used

The data extracted from the work of Knight and Sterling [6] were used to investigate the feasibility of Tsallis-based shear stress distribution in circular channels. Using the Preston pipe technique, Knight and Sterling [6] could measure shear stress distribution in different flow depths of a circular conduit with a diameter of 244 mm and a wall thickness of 3 mm. The τ was obtained based on multiple sediment layer thickness *t*, with the bed horizontally scraped to make a flat bed. Table 2 lists the main hydraulic parameters of 12 experimental cases. Note that the symbol *S*_0_ denotes the channel length slope, *Q* is the flow discharge, and *Fr* is the Froude number.

Yuen [5] measured shear stress distribution in a trapezoidal channel with different flow conditions, so these data were utilized to verify the obtained equation using Tsallis entropy. His experiments were conducted in a 21.26 m long, 0.615 m wide, and 0.365 m depth. The hydraulic parameters applied in his experimental setup are presented in Table 3.

## 5. Performance Evaluation

Five statistical evaluation criteria were adopted to assess the abilities of the Tsallis- and Shannon-based models to predict shear stress distribution in the circular channel with sediment and the trapezoidal channel. These criteria are the Root Mean Square Error (RMSE), Mean Absolute Error (MAE), Percentage of BIAS (PBIAS), the RMSE of the standard deviation of observation ratio (RSR), and Nash Sutcliffe Efficiency (NSE), respectively, which are given by these expressions:

(34) $$RMSE=\sqrt{\frac{\sum_{i=1}^{n}{\left(\tau_{ip}-\tau_{im}\right)}^{2}}{n}}$$

(35) $$MAE=\frac{1}{n}\sum_{i=1}^{n}\left|\tau_{ip}-\tau_{im}\right|$$

(36) $$RSR=\sqrt{\frac{\sum_{i=1}^{n}{\left(\tau_{ip}-\tau_{im}\right)}^{2}}{\sum_{i=1}^{n}{\left(\tau_{im}-{\bar{\tau }}_{im}\right)}^{2}}}$$

(37) $$PBIAS=100\times \left[\frac{\sum_{i=1}^{n}\left(\tau_{im}-\tau_{ip}\right)}{\sum_{i=1}^{n}\tau_{im}}\right]$$

(38) $$NSE=1-\frac{\sum_{i=1}^{n}{\left(\tau_{im}-\tau_{ip}\right)}^{2}}{\sum_{i=1}^{n}{\left(\tau_{im}-{\bar{\tau }}_{im}\right)}^{2}}$$

where $\tau_{im}$ is the measured shear stress in laboratory, $\tau_{ip}$ is shear stress values computed by models, and ${\bar{\tau }}_{im}$ and ${\bar{\tau }}_{ip}$ are the measured and predicted mean values.

According to studies of Moriasi et al. [39], Table 4 indicates the performance analysis of statistical criteria. As shown in this table, four categories are defined to evaluate the performance of models.

## 6. Results

**Figure 3:**
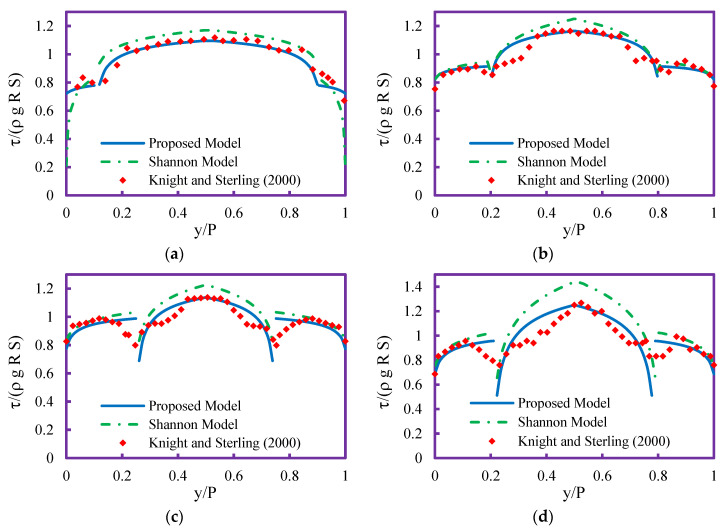
Shear stress distribution profile in circular channel with flat bed for (**a**) case 1, (**b**) case 3, (**c**) case 7, (**d**) case 8.

To investigate the performance of the proposed model in estimating shear stress distribution in circular channels with flat bed and trapezoidal channels, a comparison with the method of Sterling and Knight [25] (Shannon entropy) was done. The results of the proposed and Shannon models in predicting the shear stress distribution in circular channel with flat bed are shown in Figure 3. As seen in the figure, the Shannon model predictions of bed shear stress are overestimated. The differences between the Shannon model results and measured data in laboratory increased with increasing sediment thickness (Figure 3d). The proposed model estimated more accurate results for bed shear stress than the Shannon model. For the wall shear stress prediction, the Shannon model demonstrated a different trend of prediction. For lower flow depth, the Shannon model predictions for wall shear stress are underestimated, but with increasing flow depth, the predictions are somewhat overestimated. The proposed model performance for estimating wall shear stress is higher than the Shannon model. Neither models could predict the trend of wall shear stress in the intersection of wall and bed because the trend of both models is ascending. All of all the proposed models demonstrated higher performance than the Shannon model in estimating shear stress distribution along the whole wetted perimeter of circular channel with sediment.

**Figure 4:**
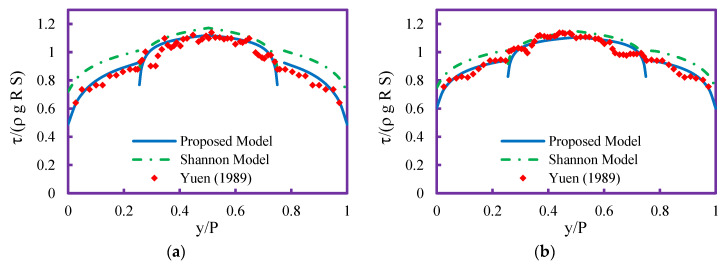

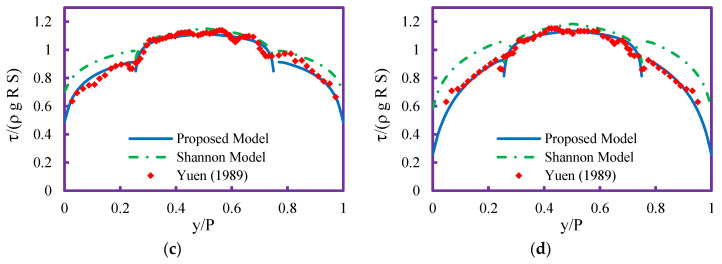
Shear stress distribution profile in trapezoidal channel for (**a**) case 17, (**b**) case 18, (**c**) case 19, (**d**) case 22.

Figure 4 shows the shear stress distribution prediction by the proposed and Shannon models in the wetted perimeter of a trapezoidal channel in different flow conditions. In all cases, the predictions of the proposed model by Sterling and Knight [25] of the wall and bed shear stress are higher than the predictions by the proposed model. The performance of the Shannon model in predicting the shear stress distribution is decreased with increasing flow depth (Figure 4d). As seen in Figure 4, both models presented good performance, but the proposed model predictions are closer to the experimental data of Yuen [5] than the Shannon model. By comparing Figure 3 and Figure 4, it can be deduced that the proposed models’ performance in estimating shear stress distribution along the wetted perimeter in trapezoidal channels is higher than a circular channel with flat bed.

Table 5 demonstrates the performance of the proposed and the Shannon-based model in estimating the shear stress distribution based on the statistical parameters. Based on the results shown in Table 5, the performance of the proposed model in estimating the shear stress distribution in trapezoidal channels with a mean MAE value of 0.0063 is higher than in the circular channel with sediment (a mean MAE value of 0.1608). Also, the proposed model could estimate shear stress in all cases better than the Shannon-based equations. The best results of estimating shear stress are for the cases 18 and 19 with RMSE values of 0.0034 and 0.0071 for the Tsallis and the Shannon models, respectively. In addition, the least satisfactory results are obtained in case 8 for both the Tsallis and the Shannon models with RSR of 0.5774 and 0.8627, respectively. However, the Tsallis model in case 8 with 0.5 < RSR < 0.6 is categorized as good performance, but in comparison with other cases, it is acknowledged that the model performance decreased in modeling the shear stress distribution in this case. The proposed model with NSE values of more than 0.93 demonstrates higher performance than the Shannon-based model in the shear stress predictions in the trapezoidal channels cross section. As shown by the results of the proposed model, the Shannon-based model performed better in estimating the shear stress values in trapezoidal channels with higher NSE values than the circular channel with flat bed, based on the lower values of NSE. The proposed model overestimates the shear stress values in cases 7, 19, and 22 due to the negative values of PBIAS of −0.0195, −0.0007, and −0.0041, respectively, and the overestimated shear stress values are obtained by the Shannon-based model in Case 17 with a PBIAS of −0.0055.

## 7. Conclusions

Since the knowledge of shear stress values could solve a significant number of engineering problems in designing open channels, in this present study, shear stress distribution is derived using Tsallis entropy. The circular and trapezoidal cross sections are common in sewers and irrigation, respectively, so the application of derived equations was tested in these sections. A new, dimensionless parameter *G* was defined to simplify the equations presented by Bonakdari et al. [26] which requires solving the complex Lagrange multipliers. The proposed equation is only related to the dimensionless parameter *G*, which can be computed from a simple equation with the knowledge of mean and maximum shear stresses. The proposed equation was verified using the credible experimental results of Knight and Sterling [6] and Yuen [5]. Also, the shear stress predictions by proposed models was compared with results of equations proposed by Sterling and Knight [25] based on Shannon entropy. According to the results, the proposed model performance in the circular channel with a flat bed for lower flow depth was higher than higher flow depth. Both the proposed and Sterling and Knight’s [25] methods showed the same performance in estimating wall and bed shear stress, the proposed model predictions values were closer to the experimental data than the Sterling and Knight [18] method. In the trapezoidal channel, the proposed model showed good performance and could predict shear stress distribution along a wetted perimeter with high precision. The proposed model showed high performance in predicting shear stress distribution and is envisaged can be used for other cross sections.

## Figures and Tables

**Table 1:** The difference in computation of *G* values.

$\overset{¯}{\tau }\;(\mathrm{pa})$	$\tau_{max}\;(\mathrm{pa})$	λ	$\lambda_{*}$	G[Equation(20)]	G[Equation(25)]	Difference
1.6350	1.8000	4.9600	−10.6850	5.0814	4.8790	0.2024
0.4752	0.5234	12.5560	−7.8562	5.0954	4.8978	0.1976
0.2242	0.2465	22.2818	−6.5571	5.1651	4.9787	0.1864
3.1000	3.4090	3.1020	−12.6280	5.1502	4.9784	0.1718
0.5824	0.6405	10.8627	−8.3102	5.1432	4.9731	0.1701
0.4249	0.4670	13.8670	−7.7230	5.1927	5.0270	0.1657
1.2230	1.0940	6.3780	−9.8240	3.8545	3.7076	0.1469
0.2943	0.3221	18.9670	−7.2327	5.4381	5.4211	0.0170
0.5216	0.5705	12.4250	−8.3823	5.4816	5.4765	0.0051
0.6246	0.6458	24.1077	−16.6096	14.9796	14.9982	−0.0186
0.6360	0.6950	10.802	−8.8600	5.5503	5.5737	−0.0234
0.5935	0.6468	11.6858	−8.8789	5.7238	5.8514	−0.1276
2.9928	3.2610	3.4788	−13.3221	5.7361	5.8698	−0.1337
0.5225	0.5675	13.3073	−8.8166	5.9733	6.2114	−0.2381

**Table 2:** The hydraulic parameters in a circular channel with flat bed.

*t/D*	Case	*h*(*mm*)	(*h*+*‘t*)/D	*S*_0_	*Q* (*Ls*^−1^)	τ_0_ (*Pa*)	*Fr*
0.25	1	20.3	0.333	0.00862	3.39	1.176	0.663
	2	36.3	0.398	0.00196	3.30	0.5447	0.656
	3	60.8	0.499	0.00196	8.00	0.8044	0.748
	4	101.5	0.666	0.00196	16.50	1.0920	0.680
	5	60.8	0.499	0.00862	18.20	3.538	1.70
	6	123.2	0.755	0.00196	22.10	1.176	0.663
0.332	7	40.7	0.499	0.009	12.00	0.967	1.960
	8	81.5	0.666	0.002	12.20	0.967	0.685
	9	114.2	0.800	0.002	22.10	1.106	0.721
0.5	10	39.5	0.666	0.009	8.40	2.571	1.4
	11	60	0.750	0.009	16.00	3.340	1.42
	12	72.2	0.800	0.009	20.00	3.645	1.33

**Table 3:** The hydraulic parameters in a trapezoidal channel.

Case	Expt. No.	*h*(*m*)	*b/h*	*S*_0_	*Q* (*Ls*^1^)	τ_0_ (*Pa*)	*Fr*
13	001	0.0300	5.000	0.001	1.3000	0.2256	0.4794
14	002	0.0375	4.000	0.001	4.650	0.2693	0.5455
15	012	0.0938	1.599	0.001	10.60	0.5398	0.5693
16	018	0.0585	0.752	0.001	1.650	0.2807	0.4553
17	022	0.0360	12.5	0.001	5.700	0.3109	0.5682
18	103	0.0440	10.23	0.001	19.40	1.4727	1.4179
19	208	0.0449	10.23	0.009	30.01	3.2305	2.1934
20	302	0.0745	2.013	0.015	32.50	6.6020	2.6236
21	408	0.044	1.000	0.023	6.500	5.2680	3.1299
22	412	0.0445	10.11	0.023	50.00	8.7577	3.5910

**Table 4:** Classification of performance of statistical parameters.

Statistical Parameters	Performance Evaluation Criteria			
	Very good	Good	Satisfactory	Unsatisfactory
RSR	0.00 < RSR < 0.50	0.50 < RSR < 0.60	0.60 < RSR < 0.70	RSR > 0.70
PBIAS	PBIAS< ± 10	± 10 ≤ PBIAS < ± 15	± 15 ≤ PBIAS < ± 25	PBIAS ≥ ± 25
NSE	NSE> 0.80	0.70 < NSE ≤ 0.80	0.50 < NSE ≤ 0.70	NSE ≤ 0.50
RMSE	The lower RMSE value, the better model performance			
MAE	The lower MAE value, the better model performance			

**Table 5:** Statistical parameters for two based entropy models.

Case No.	Proposed Model					Shannon Model				
	RMSE	MAE	RSR	PBIAS	NSE	RMSE	MAE	RSR	PBIAS	NSE
1	0.0544	0.0367	0.4025	0.0147	0.8096	0.0899	0.0645	0.6256	0.0485	0.4796
3	0.0701	0.503	0.5824	0.0330	0.6923	0.1060	0.0675	0.8409	0.0649	0.2965
7	0.0619	0.0472	0.3842	−0.0195	0.8243	0.0824	0.0530	0.4608	0.0153	0.6891
8	0.0855	0.0563	0.5774	0.0520	0.5954	0.1815	0.1408	0.8627	0.1400	−0.8213
17	0.0347	0.0133	0.2602	0.0121	0.9314	0.0565	0.0297	0.4435	−0.0055	0.818
18	0.0077	0.0029	0.0677	0.0003	0.9956	0.0537	0.0413	0.6212	0.0306	0.7842
19	0.0071	0.0031	0.0490	−0.0007	0.9977	0.0459	0.0307	0.3929	0.0266	0.9010
22	0.0139	0.0059	0.0945	−0.0041	0.9911	0.0766	0.0587	0.6532	0.0587	0.7300

## References

[1] Knight D.W., Demetriou J.D., Hamed M.E. (1984). Boundary shear in smooth rectangular channels. J. Hydraul. Eng..

[2] Seckin G., Seckin N., Yurtal R. (2006). Boundary shear stress analysis in smooth rectangular channels. Can. J. Civ. Eng..

[3] Tominaga A., Nezu I., Ezaki K., Nakagawa H. (1989). Three-dimensional turbulent structure in straight open channel flows. J. Hydraul. Res..

[4] Alhamid A. (1991). Boundary Shear Stress and Velocity Distribution in Differentially Roughened Trapezoidal Open Channels. Ph.D. Thesis.

[5] Yuen K.W.H. (1989). A Study of Boundary Shear Stress Flow Resistance and Momentum Transfer in Open Channels with Simple and Compound Trapezoidal Cross Section. Ph.D. Thesis.

[6] Knight D.W., Sterling M. (2000). Boundary shear in circular pipes running partially full. J. Hydraul. Eng..

[7] Kleijwegt R.A. (1992). On Sediment Transport in Circular Sewers with Non-cohesive Deposits. Ph.D. Thesis.

[8] Khodashenas S.R., Paquier A. (1999). A geometrical method for computing the distribution of boundary shear stress across irregular straight open channels. J. Hydraul. Res..

[9] Yang S.-Q., Lim S.-Y. (2005). Boundary shear stress distributions in trapezoidal channels. J. Hydraul. Res..

[10] Zarrati A.R., Jin Y.C., Karimpour S. (2008). Semianalytical model for shear stress distribution in simple and compound open channels. J. Hydraul. Eng..

[11] Sheikh Khozani Z., Bonakdari H., Ebtehaj I. (2017). An analysis of shear stress distribution in circular channels with sediment deposition based on Gene Expression Programming. Int. J. Sediment Res..

[12] Sheikh Khozani Z., Bonakdari H., Zaji A.H. (2018). Estimating shear stress in a rectangular channel with rough boundaries using an optimized SVM method. Neural Comput. Appl..

[13] Sheikh Khozani Z., Bonakdari H. (2016). A comparison of five different models in predicting the shear stress distribution in straight compound channels. Sci. Iran. Trans. A Civ. Eng..

[14] Afan H.A., El-shafie A., Mohtar W.H.M.W., Yaseen Z.M. (2016). Past, present and prospect of an Artificial Intelligence (AI) based model for sediment transport prediction. J. Hydrol..

[15] Wan Mohtar W.H.M., Afan H., El-Shafie A., Bong C.H.J., Ab. Ghani A. (2018). Influence of bed deposit in the prediction of incipient sediment motion in sewers using artificial neural networks. Urban Water J..

[16] Toriman E., Jaafar O., Maru R., Arfan A., Ahmar A.S., Uca (2018). Daily Suspended Sediment Discharge Prediction Using Multiple Linear Regression and Artificial Neural Network. J. Phys. Conf. Ser..

[17] Sheikh Khozani Z., Hosseinjanzadeh H., Wan Mohtar W.H.M. (2019). Shear force estimation in rough boundaries using SVR method. Appl. Water Sci..

[18] Sheikh Khozani Z., Bonakdari H., Zaji A.H. (2016). Application of a genetic algorithm in predicting the percentage of shear force carried by walls in smooth rectangular channels. Measurement.

[19] Sheikh Khozani Z., Khosravi K., Pham B.T., Kløve B., Wan Mohtar W.H.M., Yaseen Z.M. (2019). Determination of compound channel apparent shear stress: application of novel data mining models. J. Hydroinform..

[20] Sheikh Khozani Z., Bonakdari H., Ebtehaj I. (2018). An expert system for predicting shear stress distribution in circular open channels using gene expression programming. Water Sci. Eng..

[21] Sheikh Khozani Z., Bonakdari H., Zaji A.H. (2017). Estimating the shear stress distribution in circular channels based on the randomized neural network technique. Appl. Soft Comput..

[22] Chiu C.L. (1987). Entropy and probability concepts in hydraulics. J. Hydraul. Eng..

[23] Luo H., Singh V.P. (2011). Entropy Theory for Two-Dimensional Velocity Distribution. J. Hydrol. Eng..

[24] Cui H., Singh V.P. (2013). Suspended sediment concentration in open channels using Tsallis entropy. J. Hydrol. Eng..

[25] Sterling M., Knight D. (2002). An attempt at using the entropy approach to predict the transverse distribution of boundary shear stress in open channel flow. Stoch. Environ. Res. risk Assess..

[26] Bonakdari H., Sheikh Z., Tooshmalani M. (2015). Comparison between Shannon and Tsallis entropies for prediction of shear stress distribution in open channels. Stoch. Environ. Res. Risk Assess..

[27] Zhang D., Jia X., Ding H., Ye D., Thakor N.V. (2010). Application of tsallis entropy to EEG: Quantifying the presence of burst suppression after asphyxial cardiac arrest in rats. IEEE Trans. Biomed. Eng..

[28] Vila M., Bardera A., Feixas M., Sbert M. (2011). Tsallis mutual information for document classification. Entropy.

[29] Varotsos P., Sarlis N., Skordas E. (2018). Tsallis Entropy Index q and the Complexity Measure of Seismicity in Natural Time under Time Reversal before the M9 Tohoku Earthquake in 2011. Entropy.

[30] Mirauda D., De Vincenzo A., Pannone M. (2018). Simplified entropic model for the evaluation of suspended load concentration. Water.

[31] Sheikh Z., Bonakdari H. (2016). Prediction of boundary shear stress in circular and trapezoidal channels with entropy concept. Urban Water J..

[32] Sheikh Khozani Z., Bonakdari H. (2018). Formulating the shear stress distribution in circular open channels based on the Renyi entropy. Phys. A Stat. Mech. Its Appl..

[33] Zhu Z., Yu J. (2019). Estimating the Bed-Load Layer Thickness in Open Channels by Tsallis Entropy. Entropy.

[34] Bonakdari H., Tooshmalani M., Sheikh Z. (2015). Predicting shear stress distribution in rectangular channels using entropy concept. Int. J. Eng. Trans. A Basics.

[35] Tsallis C. (1988). Possible generalization of Boltzmann-Gibbs statistics. J. Stat. Phys..

[36] Jaynes E.T. (1982). On the rationale of maximum-entropy methods. Proc. IEEE.

[37] Jaynes E.T. (1957). Information theory and statistical mechanics. II. Phys. Rev..

[38] Knight D.W., Yuen K.W.H., Alhamid A.A.I., Beven K., Chatwin P.C., Millbark J. (1994). Boundary Shear Stress Distributions in Open Channel Flow in Physical Mechanisms of Mixing and Transport in the Environment.

[39] Moriasi D.N., Arnold J.G., Van Liew M.W., Binger R.L., Harmel R.D., Veith T.L. (2007). Model evaluation guidelines for systematic quantification of accuracy in watershed simulations. Trans. ASABE.

